# Targeting arrhythmogenic macrophages: lessons learned from arrhythmogenic cardiomyopathy

**DOI:** 10.1172/JCI180482

**Published:** 2024-05-15

**Authors:** Thassio Mesquita, Eugenio Cingolani

**Affiliations:** Smidt Heart Institute, Cedars-Sinai Medical Center, Los Angeles, California, USA.

## Abstract

Arrhythmogenic cardiomyopathy (ACM) is an inherited cardiac condition characterized by cardiac remodeling and life-threatening ventricular arrhythmias. In this issue of the *JCI*, Chelko, Penna, and colleagues mechanistically addressed the intricate contribution of immune-mediated injury in ACM pathogenesis. Inhibition of nuclear factor κ-B (NF-κB) and infiltration of monocyte-derived macrophages expressing C-C motif chemokine receptor-2 (CCR2) alleviated the phenotypic ACM features (i.e., fibrofatty replacement, contractile dysfunction, and ventricular arrhythmias) in desmoglein 2–mutant (*Dsg2*^mut/mut^) mice. These findings pave the way for efficacious and targetable immune therapy for patients with ACM.

## Arrhythmogenic cardiomyopathy pathophysiology

Arrhythmogenic cardiomyopathy (ACM) is an inherited cardiac condition that is not secondary to ischemic, hypertensive, or valvular heart disease (1). Clinically, patients with ACM present ventricular arrhythmias that can lead to sudden cardiac death. Arrhythmogenic right ventricular cardiomyopathy (ARVC), the most extensively characterized form of the disease, is mostly caused by autosomal dominant inheritance of genes encoding desmosomal proteins (1). Among the pathogenic variants, mutations on plakophilin 2 (*PKP2*) are reported to account for 20%–46% of ARVC cases, whereas desmoplakin and desmoglein 2 (*DSG2*) account for 10% each, desmocollin 2 for 5%, and plakoglobin for less than 1% (1–4). Desmosomes are intercellular junctions that communicate mechanical stress between cardiomyocytes; their interaction with other intercalated disc proteins forming a single functional unit facilitates cell-to-cell coupling. Hence, a mutation on a single gene is generally sufficient to disrupt cell-to-cell coupling leading to inflammation, the typical fibrofatty tissue replacement, impaired contractility, and electrical instability in ACM.

## Arrhythmogenic cardiomyopathy as a possible inflammatory disorder

Inflammation has been a recurrent theme in ACM pathophysiology (5), with reports of multifocal patchy myocarditis in both ventricles of patients with ARVC (6, 7). The most accepted hypothesis is that cardiomyocyte death generates signals to activate cardiac resident immune cells, eliciting the recruitment of leukocytes to the heart. Although the accumulation of inflammatory cells is necessary to scavenge dead myocytes, an uncoordinated transition to the reparative phase may lead to maladaptive remodeling, forming the cellular basis of arrhythmogenic substrates in ACM (8, 9). Advances in platforms for single-cell sequencing have fueled the field of cardioimmunology by identifying unanticipated roles of other cardiac cells. In this context, macrophages have been recognized to be abundant and heterogeneous within the heart, playing canonical and noncanonical roles. Despite the wealth of knowledge on ACM pathophysiology, little has been known about the specific immune cell-mediated inflammation until now.

## Mechanisms of immune-mediated injury in arrhythmogenic cardiomyopathy

In this issue of the *JCI*, Chelko, Penna, and colleagues (10) elegantly addressed this knowledge gap by identifying a functional subset of inflammatory and proarrhythmic macrophages in a murine model of ACM. Previous studies revealed that *Dsg2*-mutant (*Dsg2^mut/mut^*) mice have characteristics that resemble clinical features of patients with ACM, including fibrofatty replacement, contractile dysfunction, ventricular arrhythmias (i.e., premature ventricular contractions [PVC] and ventricular tachycardia [VT]), exacerbated phenotype with exercise, and inflammation (11–14). Further, activation of nuclear factor κ-B (NF-κB) is recognized as a mediator of inflammation in *Dsg2^mut/mut^* mice, while its inhibition with Bay 11-7082 remediates key ACM features (11).

**Figure 1:**
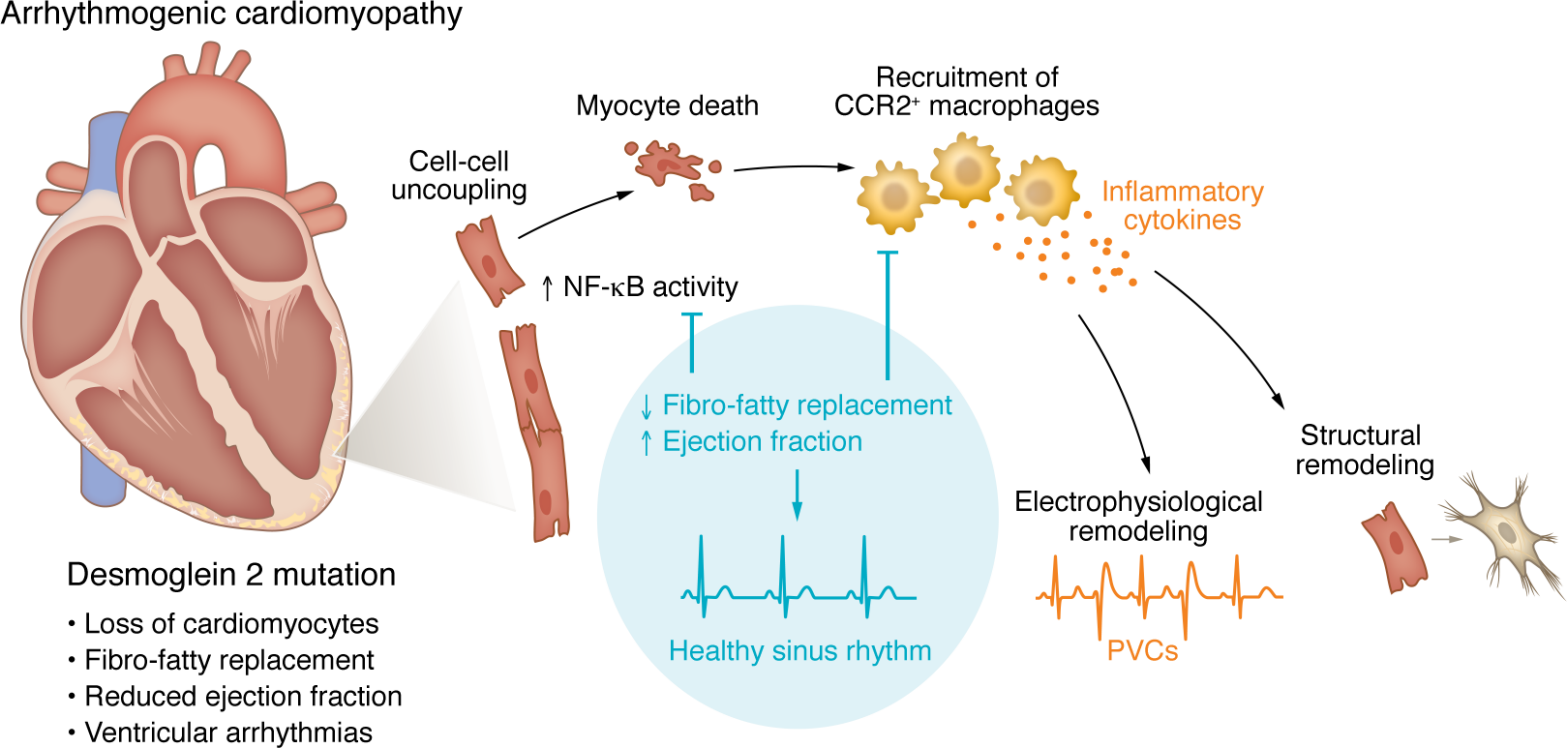
Macrophages contribute to proarrhythmic mechanisms in ACM. Phenotypic features of the *DSG2* pathogenic variant include fibro-fatty replacement, impaired contractility, and ventricular arrhythmias. Mutation of the *Dsg2* gene in mice leads to the disruption of myocyte-myocyte interaction at the intercalated disc and damages to the cardiomyocyte cell membrane, which activates NF-κB and leads to cell death. NF-κB signaling is a major determinant of monocyte-derived macrophage recruitment, particularly the ones expressing CCR2. The release of proinflammatory cytokines by CCR2^+^ macrophages may alter cardiac electrophysiology by affecting ionic remodeling in cardiomyocytes and extracellular matrix remodeling. Inhibition of NF-κB signaling and the subsequent prevention of CCR2^+^ macrophage infiltration limits the phenotypic ACM features in *Dsg2^mut/mut^* mice.

Taking advantage of the opportunity to study temporal changes in cardiac immune populations of the ACM animal model, Chelko, Penna, and colleagues (10) showed that monocyte-derived macrophages expressing C-C motif chemokine receptor-2 (CCR2) infiltrated the heart as early as four weeks of age and persisted at elevated levels until the study endpoint (16 weeks). To define whether infiltrated CCR2^+^ macrophages are mechanistically linked with ACM pathogenesis, a *Dsg2^mut/mut^* mouse lacking *Ccr2* (*Dsg2^mut/mut^/Ccr2^–/–^*) was generated. The inability to mobilize CCR2^+^ macrophages to the hearts of *Dsg2^mut/mut^* mice prevented the fibrotic remodeling and mitigated the increased PVC burden. Similar observations were found in *Dsg2^mut/mut^* mice expressing a cardiac-specific dominant-negative of the inhibitoar of κBα, suggesting that activation of NF-κB signaling and recruitment of CCR2^+^ macrophages are interconnected (Figure 1). A parallel study by the authors confirmed the expansion of CCR2^+^ cells and NF-κB activation in hearts from patients with ACM compared with age-matched controls with no history of heart disease (15).

The combination of single nuclei RNA sequencing (snRNA-Seq) and cellular indexing of transcriptomes and epitomes sequencing (CITE-Seq) has furnished a detailed transcriptional landscape at the cellular level. This approach revealed that CCR2^+^-expressing cells serve as the primary cellular sources of inflammatory cytokines in ACM hearts. An additional key finding of Chelko et al. (10) is that the transcriptional signatures of fibroblast activation, periostin-expressing fibroblasts, were dependent on CCR2^+^ macrophages. Moreover, using a clinically realistic experimental design, *Dsg2^mut/mut^* mice with established ACM phenotype treated with an NF-κB inhibitor presented notable benefits with additive effects seen in *Dsg2^mut/mut^/CCR2^–/–^* mice.

## Perspectives and future directions

While Chelko et al. (10) present stimulating insights into ACM pathophysiology, it is crucial to reflect on these findings while considering limitations and future investigations. Pathogenic desmosomal variants have low, variable, and age-dependent penetrance (1–4). Thus, homozygous *Dsg2^mut/mut^* mice display a premature ACM phenotype with first signs of inflammation as early as two weeks of life. Although this ACM model may provide an exaggerated representation of human disease, the beneficial effects of NF-κB inhibition in such a severe phenotype are encouraging. In addition, despite the remarkable antifibrotic effects, the restoration of ejection fraction remained suboptimal. The exception applies to the inhibition of NF-κB signaling in *Dsg2^mut/mut^/Ccr2^–/–^* mice, suggesting that other potential mechanisms might contribute to changes in contractility. As acknowledged by the authors, other inflammatory cells may also participate in the pathogenesis of ACM. Therefore, the public access to the large transcriptional datasets at the cellular level granted by the authors may propel investigational directions beyond macrophages.

From an electrophysiological perspective, the precise mechanisms by which CCR2^+^ macrophages and other leukocytes alter the cardiac excitability remain to be elucidated. Paracrine signaling mediated by the secretion of proinflammatory cytokines is a likely explanation, as indicated in Chelko et al. (10). Leukocyte-released cytokines are recognized for their role in influencing the phenoconversion of cardiac fibroblasts into myofibroblasts, which, in turn, leads to conduction slowing and heterogeneity, favoring arrhythmogenic substrates. As the field continues to evolve, the crosstalk between leukocytes and cardiomyocytes, either by paracrine or electrotonic coupling, deserves more attention. In the context of ACM, cardiomyocyte injury and death are likely the primary triggers of local inflammation, which, in turn, can promote additional ectopic arrhythmic activity from surrounding myocytes. Proinflammatory cytokines prolong cardiomyocyte action potential duration by decreasing repolarizing potassium currents, and can favor alterations in other membrane-bound ion channels and intracellular calcium handling molecules. Future investigations are necessary to understand the multifaceted roles of leukocytes in ACM and other cardiac conditions that exhibit arrhythmogenic substrates.

Arrhythmogenic disease-causing genetic mutations are mostly expressed in cardiomyocytes, where myocyte-specific gene therapy has been tailored to correct the primary source of the disease. A series of independent studies have recently demonstrated promising outcomes using gene replacement therapy to overexpress PKP2 in preclinical models of ACM (16–18). The consolidation of these high expectations will be validated as three FDA-approved adeno-associated virus–mediated gene therapies for PKP2 advance into phase 1 clinical trials. Despite the translational potential of gene therapy in ACM, safety and durability remain common concerns (19). Thus, similar concerns and precautions should be considered for immune cell-targeted therapies. For instance, cases of cardiotoxicity and arrhythmias have been reported with immune checkpoint inhibitor therapy. The research by Chelko et al. (10) sheds light on the future of ACM therapies: with continuous developments in gene- and immune-based therapies, it is conceivable that targeting inflammatory macrophages may further enhance the effectiveness of gene therapy for ACM or serve as an alternative for patients with gene therapy-refractory ACM.

## Version 1 05/15/2024

Electronic publication
